# CVD Risk Stratification in the PCSK9 Era: Is There a Role for LDL Subfractions?

**DOI:** 10.3390/diseases6020045

**Published:** 2018-05-27

**Authors:** Christian Abendstein Kjellmo, Anders Hovland, Knut Tore Lappegård

**Affiliations:** 1 Division of Internal Medicine, Nordland Hospital, N-8092 Bodø, Norway; anders.w.hovland@gmail.com (A.H.); knut.tore.lappegard@gmail.com (K.T.L.); 2 Department of Clinical Medicine, University of Tromsø, N-9037 Tromsø, Norway

**Keywords:** PCSK9, proprotein convertase subtilisin/kexin type 9, LDL subfractions, sdLDL, cardiovascular disease, risk stratification

## Abstract

Proprotein convertase subtilisin/kexin type 9 (PCSK9) inhibitors reduce the risk of cardiovascular events and all-cause mortality in patients at high risk of cardiovascular disease (CVD). Due to high costs and unknown long-term adverse effects, critical evaluation of patients considered for PCSK9 inhibitors is important. It has been proposed that measuring low-density lipoprotein (LDL) subfractions, or LDL particle numbers (LDL-P), could be of value in CVD risk assessment and may identify patients at high risk of CVD. This review evaluates the evidence for the use of LDL subfractions, or LDL-P, when assessing CVD risk in patients for whom PCSK9 inhibitors are considered as a lipid-lowering therapy. Numerous methods for measuring LDL subfractions and LDL-P are available, but several factors limit their availability. A lack of standardization makes comparison between the different methods challenging. Longitudinal population-based studies have found an independent association between different LDL subfractions, LDL-P, and an increased risk of cardiovascular events, but definitive evidence that these measurements add predictive value to the standard risk markers is lacking. No studies have proven that these measurements improve clinical outcomes. PCSK9 inhibitors seem to be effective at lowering all LDL subfractions and LDL-P, but any evidence that measuring LDL subfractions and LDL-P yield clinically useful information is lacking. Such analyses are currently not recommended when considering whether to initiate PCKS9 inhibitors in patients at risk of CVD.

## 1. Introduction

Despite major advances in the prevention and treatment of cardiovascular diseases (CVD) over the last few decades, CVD continues to be the leading global cause of death and morbidity [1]. Several different guidelines for CVD prevention are available and the recommended overall strategy is the targeting of modifiable risk factors in high risk patients [2,3].

Of the multiple modifiable risk factors associated with cardiovascular disease [4], low-density lipoprotein (LDL) is the most intensively studied and a causal relationship with the development of CVD has been established [5]. Managing LDL-related risk is emphasized in all CVD prevention guidelines by recommending lipid-lowering therapy, usually statins, to all patients for secondary prevention, and to high-risk patients for primary prevention [6]. The guidelines for CVD prevention are not unified in their recommendations on what lipoprotein measurement to use in risk assessment and as a target of therapy [2,3,7]. Non-high-density lipoprotein cholesterol (non-HDL-C) is the lipoprotein measurement recommended for risk assessment in most guidelines, as it reflects all the cholesterol mass with atherogenic potential and avoids the biases that might arise when using the Friedewald formula to calculate LDL cholesterol (LDL-C) [8]. LDL-C is still the most widely-recommended primary target of therapy. Both metrics are included in the standard lipid panel, which is readily available at most clinical laboratories. Despite its central role in CVD pathophysiology, the value of both non-HDL-C and LDL-C in CVD risk stratification is limited as a significant proportion of patients who develop CVD have levels within the “normal” range [9]. Due to this, there has been intensive research into whether different advanced lipoprotein testing methods may improve cardiovascular risk prediction.

LDL-C is a measure of the total cholesterol content in LDL particles. LDL-C and LDL particle number (LDL-P) is usually highly correlated [10]. Under certain circumstances, notably in patients with diabetes, metabolic syndrome, or hypertriglyceridemia, LDL-C and LDL-P can become discordant due to the predominance of small dense cholesterol-depleted LDL-particles (sdLDL) [11]. In these patients, LDL-C might not accurately reflect the LDL-related risk for cardiovascular disease, and studies have shown that LDL-P has a stronger association with CVD risk compared to LDL-C in patients with discordant levels of LDL-C and LDL-P [10,11]. Due to this fact, it has been proposed that measuring subfractions or the particle number of LDL, might enhance CVD risk assessment in the general population and detect residual risk in patients already receiving lipid-lowering therapy.

Recent advances in lipid lowering therapies, with the development of proprotein convertase subtilisin/kexin type 9 (PCSK9) inhibitors [12], has reignited attention on CVD-risk stratification. Clinicians now have the tools to reduce LDL to very low levels, but the costs are significant and potential side effects have only been evaluated in relatively short-term studies. For this reason, PCKS9 inhibitors are currently only recommended to patients at a very high risk, such as patients with familial hypercholesterolemia (FH), statin-intolerant patients in secondary prevention, or in secondary prevention for patients with high residual risk [13,14].

In this review, we sought to evaluate the evidence for the use of LDL subfractions in CVD risk assessment in general, and to assess if the available methods for LDL subfractioning could be of value for clinicians in the decision of whether to initiate PCKS9 therapy in patients.

## 2. LDL Subfractions—And How to Separate Them

**Figure 1:**
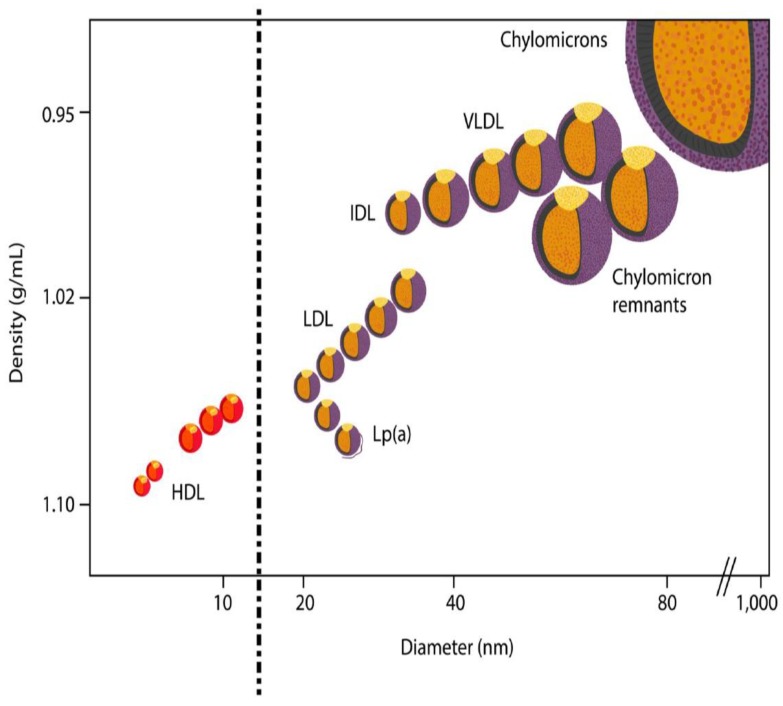
Relative size and density of the major plasma lipoproteins and their subfractions.

LDLs are broadly defined as lipoproteins with a density in the range of 1.019–1.063 g/mL, and each particle containing one apolipoprotein B (apoB) molecule (Figure 1). LDL particles are heterogeneous with respect to size, density, and composition, and can be separated based on various physicochemical properties depending on the protein purification technique used. The methods commonly used in the published literature on LDL subfractions are usually based on gel electrophoresis (GE), nuclear magnetic resonance (NMR), ultracentrifugation (UC), or ion mobility (IM), but several other methods are also available [15,16]. 

The accessibility of these methods is limited. GE, NMR, UC, and IM are technically demanding, expensive, and too labor-intensive for routine use in clinical laboratories. Most methods used in published studies are patented laboratory developed tests, which are only available at one laboratory or a limited number of laboratories. Two exceptions include a simple and less-expensive tube gel electrophoresis-system (Lipoprint^®^) [17] and a homogeneous assay (HA) adaptable to autoanalyzers [16].

The different methods use various terms to describe LDL particles, their distribution, and characteristics, including: LDL subfractions, LDL subclasses, LDL particle and subfraction concentration, LDL particle diameter, LDL peak diameter, and others [18,19,20]. These terms describe overlapping attributes of the LDL particles. The potential for confusion is significant, and in this review we will use the generic term LDL subfractions. LDL particle number (LDL-P) is a measurement of the total number of LDL particles across all subfractions, but will be included in this review as NMR and IM methods report it. ApoB is another indirect measurement of LDL-P and the ratio between serum cholesterol and apoB has been proposed as a metric to determine the prevalence of sdLDL [21]. However, apoB will not be included in this review as apoB is not reported by the various methods that determine LDL subfractions.

The recognition of the different LDL subfractions has led to the description of two distinct patterns of phenotypes that are reported by most LDL subfractioning methods available today [22]: Phenotype “A”, with a predominance of large buoyant LDL-particles and phenotype “B”, with a predominance of small, dense LDL particles, which is suggested as a more atherogenic phenotype. 

## 3. Comparability of Methods for LDL Subfractioning

Presently, there is no method for the determination of LDL subfractions that is considered a reference or a gold standard. The different methods commonly available (NMR, UC, GE, and IM) separate LDL particles based on different characteristics that are not directly comparable; GE separates LDL particles based on size and charge, UC based on density, NMR measures methyl group signals from lipoprotein particles and calculates the LDL particle number and size, while IM separates lipoprotein particles using gas-phase electrophoresis and directly counting the size-separated particles.

In 2009, Chung et al. [23] published a comprehensive review of the comparability between several different methods for LDL subfractioning, including UC, NMR, and segmented gradient GE (sGGE). This review raised several issues. These methods separate LDL particles based on completely different physicochemical properties, and, consequently, it is not possible to determine whether the different methods are measuring the same subfractions. Different thresholds of LDL size and particle number are used to describe LDL particle distributions and LDL phenotypes, further obfuscating a reasonable comparison of results between methods. Furthermore, the wide range of agreement between the methods reported (7–94% concordance for classifying LDL phenotypes) was suggested to be due to a lack of standardization. Lastly, no study has compared the diagnostic accuracy, efficacy, or clinical value of these methods to individuals’ clinical outcomes.

Chung et al. concluded that, to permit an accurate description of the similarities and differences among the different LDL subfractioning methods, a reference material, accepted as appropriate, accurate and reliable, needed to be developed. After the establishment of a consensus reference method, epidemiological studies and clinical trials are required to examine the clinical value of LDL subfractioning to predict the individual patients’ clinical outcomes [23].

Since 2009, no reference material, nor a consensus reference method, has been established. IM has been introduced and has been used in the larger population-based studies [24,25], adding to the complexity of LDL subfractioning. A few studies, published since 2009, have looked at the comparability between different methods. Two studies have reported high rates of correlation, including a 98% agreement between IM and sGGE [26,27], but as a healthy study population was used the results need to be confirmed in a population relevant for CVD risk assessment. Williams et al. reported that both sGGE, NMR, and IM confirmed the association between small LDL particles and greater atherosclerotic progression, but the correlations between the methods varied significantly [28].

In contrast, international standards exist for assays measuring HDL-C, total cholesterol, LDL-C, apoB and apolipoprotein A1, and Lipoprotein(a). [29]

## 4. The Atherogenicity of Small, Dense LDL Particles 

The first step in the initiation of atherosclerotic disease is the subendothelial retention of apoB-containing lipoproteins, predominantly LDL, in the arterial wall [30]. Elevated levels of apoB-containing lipoproteins in the blood increases the risk of atherosclerotic disease, in a dose-dependent matter [31]. One possible explanation for increased CVD risk associated with elevated levels of sdLDL or LDL-P could be that these measures more accurately reflect the number of apoB-containing lipoproteins compared to LDL-C. 

There is also evidence indicating that sdLDL-particles have an increased atherogenic potential when compared to larger LDL particles [32]. The circulation time of sdLDLs is proposed to be longer, compared to larger LDL-particles, due to their impaired interaction with the LDL-receptor (LDLR) [33]. An increased susceptibility of sdLDL to undergo atherogenic modification in blood plasma, such as desialyation, glycation, and oxidation, has also been reported [34,35] and in-vitro studies have shown that sdLDL particles are more avidly taken up by macrophages, have a greater propensity for transport into the artery wall, and have a greater binding potential to proteoglycans in the artery wall [36,37].

## 5. LDL Subfractions and Associations with Increased CVD Risk

Ip et al. published a systematic review of associations between LDL subfractions and cardiovascular outcomes in 2009 [38]. Most trials (37 of 52) found statistically significant associations between LDL subfraction size, number or phenotype, and cardiovascular outcomes. However, only 26 analyses were adjusted for standard lipids and just 12 of the adjusted studies found significant associations with incident cardiovascular disease, including 4 of the 14 trials analysing LDL size and sdLDL, and 8 of the 12 trials analysing LDL number and phenotype. Several important limitations were noted, including the lack of comparability between the various methods used to measure LDL subfractions and a heterogeneity in the adjustment and small sample sizes. The review concluded that there is a potential association between small LDL subfractions and incident cardiovascular disease, but there is not enough data to support their value as an independent risk factor for clinical use. Standardization of LDL subfractions measures is needed and studies need to confirm that treatment based on LDL subfraction testing is beneficial.

**Figure 2:**
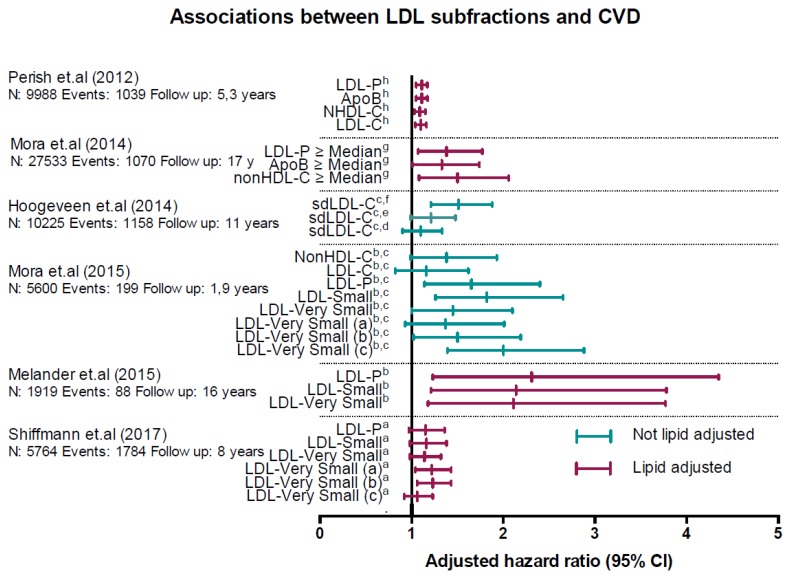
Associations between low-density lipoprotein (LDL) subfractions and cardiovascular disease (CVD) reported in prospective population-based studies published after 2009 and adjusted for confounding factors, such as age, smoking, gender, hypertension, etc. ^a^ Top vs. bottom quartile analysis. ^b^ Top vs. bottom tertile analysis. ^c^ Not significant in a model adjusted for lipids. ^d^ 1st vs. 2nd quartile analysis. ^e^ 1st vs. 3rd quartile analysis. ^f^ 1st vs. 4th quartile analysis. ^g^ Associations with CVD in patients with discordant levels of LDL particle number (LDL-P), apolipoprotein B (apoB), non-high density lipoprotein cholesterol (non-HDL-C) (≥median), and LDL cholesterol (LDL-C) (<median). ^h^ Hazard ratio (95% CI) pr. 1 SD higher. Hazard ratios for the outcome “major occlusive event” in the placebo arm are depicted in the graph.

Since 2009, several large population-based longitudinal studies have investigated the association between various LDL subfractions and cardiovascular outcomes (Figure 2).

Mora et al. (2015) [24] published a study based on clinical data and blood samples from the JUPITER-study [39]; a large, randomized prospective trial of rosuvastatin for primary CVD prevention in patients with LDL-C levels less than 130 mg/dL (<3.34 mmol/L) and elevated hsCRP. IM was used to determine the LDL subfractions. They included 11,186 participants, 5600 in the placebo arm, and 199 primary CVD events were recorded in the placebo group. Mean follow-up time was only 1.9 years as the trial was terminated early. In the placebo-arm of the study, several of the smaller LDL subfractions showed significant associations with an increased risk for incident CVD in a top vs. bottom tertile analysis, which used a model that did not adjust for standard lipids (Figure 2). In a fully adjusted model including standard lipids, only one of the smaller LDL subfractions (LDL-Very small (c)) remained significantly associated with an increased CVD risk.

Shiffman et al. (2017) [25] and Melander et al. (2015) [40] published studies based on data and blood samples from the Malmö Prevention Project (MPP), a large population-based prospective study. Shiffman et al. included 5764 participants without CVD as a baseline, and recorded 1784 CVD events over a mean follow-up time of 8.05 years. Melander et al. included 1919 participants who were not candidates for statin therapy, as defined in the 2013 American Heart Association/American College of Cardiology guidelines for the treatment of cholesterol to reduce CVD risk, and, thus, were at low risk for a CVD event. During a mean follow-up of 16.2 years, they recorded 88 CVD events (4.8%). Both studies used IM as the method for the determination of LDL subfractions.

Shiffman et al. reported that two of the five Small or Very Small LDL-subfractions were significantly associated with an increased risk for a CVD event in a top vs. bottom quartile analysis using a fully, lipid adjusted model. (Figure 2). LDL-P was not associated with an increased CVD risk. In a subgroup analysis of patients with low to intermediate risk, LDL-P and the LDL-Small subfraction was significantly associated with an increased risk, while in participants at very high risk (>20% 10-year risk), LDL-P and four of the five LDL subfractions were significantly associated with an increased risk.

Melander et al. reported that the LDL-P and the LDL-Small subfractions were associated with an increased risk of incident CVD in a fully, lipid adjusted, top vs. bottom tertile analysis, while the other four LDL-Very Small subfractions were not (Figure 2).

Hoogeveen et al. (2014) [41] published a study based on data and blood samples from the Atherosclerosis Risk in Communities (ARIC) study, a large population-based prospective study. They included 10,225 participants without prevalent CVD. Mean follow-up time was 11 years and incident CVD developed in 1158 participants. LDL subfractions were determined by a homogenous assay that quantified small, dense LDL-C (sdLDL-C). [16]. sdLDL-C was significantly associated with incident CVD in a top vs. bottom quartile analysis using a model that did not adjust for standard lipids (Figure 2), In a fully adjusted model that included standard lipids sdLDL-C was not associated with an increased risk. The cumulative incidence of CVD was higher in participants with low LDL-C and discordantly higher sdLDL-C (10.9%), compared to those with low sdLDL-C and discordantly higher LDL-C (7.9%). However, similar analyses using non-HDL-C, the recommended lipid metric for CVD risk assessment by most guidelines, was not reported.

Mora et al. (2014) [11] published a study based on the Women’s Health Study, a large prospective population-based study. Participants were women aged >45 years and were free of cardiovascular disease at admission. This study included 27,533 participants, with a mean follow-up time of 17.2 years. During the follow-up period 1070 CVD-events occurred. The aim of this study was to determine the prevalence of and long-term prognosis for cardiovascular events in participants with discordant levels of LDL-C compared with LDL-P, non-HDL-C (NHDL-C), and apoB. LDL-P was determined using NMR. Among the women with below-median LDL-C and discordantly high NHDL-C, apoB, and LDL-P above the median, LDL-C underestimated the risk of cardiovascular events by 30–50% (Figure 2), but, importantly, LDL-P was not found to be superior to NHDH-C or apoB. This is in line with a 2009 study based on the same material [11].

Parish et al. (2012) [42] published a study based on the Heart Protection Study, a randomized controlled trial that included 20536 participants, between the ages of 40 and 80 years, who received either 40mg of simvastatin or a placebo once daily, with a mean follow up time of 5.3 years. Using baseline blood samples, they evaluated the associations between a variety of lipoprotein metrics, including LDL subfractions, determined by NMR, and cardiovascular outcomes. Primary endpoints (occlusive coronary events, revascularization, other cardiac events, and stroke) were equally strongly associated with LDL-C, apoB, NHDL-C, and LDL-P (Figure 2).

Overall, these studies seem to support an independent association between small LDL subfractions, or LDL-P, and hard cardiovascular outcomes, but inconsistencies between the studies should be noted and definitive evidence that LDL subfractions add predictive value to the established standard risk markers is lacking.

## 6. LDL Subfractions and PCSK9 Inhibitors

PCSK9 inhibitors reduce serum levels of LDL by blocking the degradation of the LDL receptor (LDLR) in hepatocytes and, thus, increasing the number of LDLRs on the cell surface, increasing LDL clearance in the liver [43]. Three major randomized trials have shown that PCSK9 inhibitors reduce the risk of cardiovascular events (FOURIER [44] and SPIRE-2 [45]) and all-cause mortality (ODYSSEY Outcomes—presented at ACC 2018, not published) in high-risk patients. The drugs are well tolerated and adverse effects are minimal, including no indications of adverse neurocognitive effects in patients with very low LDL-C levels. However, long-term data are needed [46].

Based on these results, PCSK9 inhibitors have now been included in CVD prevention guidelines [13,14] as a lipid lowering therapy to be considered for patients at very high risk of CVD. Very high risk patients are usually defined as patients with familial hypercholesterolemia (FH), in secondary prevention for patients with high residual risk after statin therapy, or patients who do not tolerate appropriate doses of statins. Currently, individuals without FH in primary prevention do not fulfil cost-effectiveness criteria due to the high costs of these new drugs [47,48].

Pre-clinical trials have reported an independent association between plasma levels of PCSK9 and small LDL subfractions in patients with established CVD [49,50] and abdominally obese dyslipidemic men [51], but not in healthy subjects [52], suggesting that plasma levels of PCSK9 could be related to the metabolism of small LDL subfractions in patients at risk of CVD. An impaired affinity of sdLDL to LDLR [33] could suggest that PCSK9 inhibitors are less efficient in reducing the small LDL particles compared to the larger LDL particles. As the PCSK9 inhibitors have just recently been introduced, a limited number of clinical studies evaluating the effects of PCSK9 inhibitors on LDL subfractions have been published. Baruch et al. published a study on the effects of RG7652, a novel PCSK9 inhibitor, on LDL subfractions evaluated by NMR in a randomized trial including 248 patients [53]. In this trial, they demonstrated reductions in LDL-C of up to 52.4%, which is comparable to other PCSK9 inhibitors that are already available. Both the small and large LDL-particles were reduced significantly at the optimal dose of RG7652, but the median percentage change was lower for the smaller LDL-particles compared to the larger particles (−43% vs. −81% from baseline), and 11 of the 45 patients showed an increase in the small LDL particles at the end of the study. Two smaller studies on the PCSK9 inhibitors, alirocumab and evolocumab, also reported a proportionally larger reduction in the large LDL particles compared to the small LDL particles, but significant reductions nonetheless [54,55]. In a pilot study of three patients with FH, who converted from lipid apheresis to evolocumab, our group reported reductions of all LDL subfractions [56]. For comparison, studies evaluating the effects of statins on LDL subfractions are more discordant, with no changes [57,58], significant decreases [59,60,61,62], and also slight increases [63] of LDL subfraction metrics being reported.

To summarize, PCSK9 inhibitors seem to be effective at lowering all LDL subfractions, but with a trend towards a more efficient lowering of the larger LDL subfractions.

## 7. Conclusions

PCKS9 inhibitors have been proven to reduce the risk of cardiovascular events and all-cause mortality in patients at high risk of CVD, with minimal adverse effects reported in short-term trials. With these new drugs, physicians now have another powerful tool to aggressively target and lower LDL-C, a causal agent in the development of atherosclerotic cardiovascular disease. As these drugs come at a considerable cost, and with long-term adverse effects unknown, it is important to critically evaluate when patients should be considered for PCKS9 inhibitors. Current CVD prevention guidelines have stated that patients at very high risk of CVD should be considered for PCSK9 inhibitors.

It has been proposed that measuring LDL subfractions, specifically small dense LDL or LDL-P, could improve CVD risk assessment and identify patients at high risk of CVD. In-vitro studies have indicated that small, dense LDLs are more atherogenic than larger LDL particles. Several methods for the determination of LDL subfractions and LDL-P are used in the published literature, however, several factors limit their availability. Furthermore, a lack of standardization makes comparison between these methods challenging. As these methods separate LDL particles based on completely different physicochemical properties, it is not possible to determine whether the different methods are measuring the same subfractions.

Longitudinal population studies indicate that increased levels of small dense LDL, or increased LDL-P, are independently associated with an increased risk of cardiovascular disease. However, definitive evidence that LDL subfractions, or LDL-P, add predictive value to the standard risk markers, which are readily available at most clinical laboratories, is lacking. No studies have shown that these measurements improve clinical outcomes. Based on the 2009 American Heart Association (AHA) framework for novel risk markers [64], a new risk marker should predict future outcomes in prospective studies, add predictive information to established risk markers, improve clinical outcomes, and be determined as cost-effective when compared to established risk markers. This framework sets a high standard for novel risk markers and should be considered a guideline. However, evidence that the novel risk marker adds predictive value to an established marker is of critical importance.

Despite indications that PCSK9 inhibitors are effective at lowering sdLDL and LDL-P, important pieces of evidence proving that LDL subfractions and LDL-P yield clinically useful information is lacking, and these measurements are not recommended in the evaluation of whether to initiate PCKS9 inhibitors in patients at risk of CVD.

In order to use analysis of LDL subfractions in clinical risk evaluation, standardization of methods and a general agreement regarding which fractions to include in such an assessment is required.
